# The effects of rhein on D-GalN/LPS-induced acute liver injury in mice: Results from gut microbiome-metabolomics and host transcriptome analysis

**DOI:** 10.3389/fimmu.2022.971409

**Published:** 2022-10-27

**Authors:** Shuhui Liu, Ruiying Yin, Ziwei Yang, Feili Wei, Jianhua Hu

**Affiliations:** ^1^ Beijing Youan Hospital, Capital Medical University, Beijing, China; ^2^ Beijing Institute of Hepatology, Beijing Youan Hospital, Capital Medical University, Beijing, China

**Keywords:** rhein, acute liver injury, gut microbiota, metabolomics, transcriptomics

## Abstract

**Background:**

Rhubarb is an important traditional Chinese medicine, and rhein is one of its most important active ingredients. Studies have found that rhein can improve ulcerative colitis by regulating gut microbes, but there are few reports on its effects on liver diseases. Therefore, this study aims to investigate these effects and underlying mechanisms.

**Methods:**

Mice were given rhein (100 mg/kg), with both a normal control group and a model group receiving the same amount of normal saline for one week. Acute liver injury was induced in mice by intraperitoneal injection of D-GalN (800 mg/kg)/LPS (10 ug/kg). Samples (blood, liver, and stool) were then collected and assessed for histological lesions and used for 16S rRNA gene sequencing, high-performance liquid chromatography-mass spectrometry (LC-MS) and RNA-seq analysis.

**Results:**

The levels of ALT and AST in the Model group were abnormal higher compared to the normal control group, and the levels of ALT and AST were significantly relieved in the rhein group. Hepatic HE staining showed that the degree of liver injury in the rhein group was lighter than that in the model group, and microbiological results showed that norank_o:Clostridia_UCG-014, Lachnoclostridium, and Roseburia were more abundant in the model group compared to the normal control group. Notably, the rhein treatment group showed reshaped disturbance of intestinal microbial community by D-GalN/LPS and these mice also had higher levels of Verrucomicrobia, Akkermansiaceae and Bacteroidetes. Additionally, There were multiple metabolites that were significantly different between the normal control group and the model group, such as L-α-amino acid, ofloxacin-N-oxide, 1-hydroxy-1,3-diphenylpropan-2-one,and L-4-hydroxyglutamate semialdehyde, but that returned to normal levels after rhein treatment. The gene expression level in the model group also changed significantly, various genes such as Cxcl2, S100a9, Tnf, Ereg, and IL-10 were up-regulated, while Mfsd2a and Bhlhe41 were down-regulated, which were recovered after rhein treatment.

**Conclusion:**

Overall, our results show that rhein alleviated D-GalN/LPS-induced acute liver injury in mice. It may help modulate gut microbiota in mice, thereby changing metabolism in the intestine. Meanwhile, rhein also may help regulate genes expression level to alleviate D-GalN/LPS-induced acute liver injury.

## Introduction

Rhein is an anthraquinone compound extracted from the rhubarb plant. Multiple studies have found rhein to have various biological effects such as anti-inflammatory (1), lipid-lowering (2), antioxidant (3), and anti-tumor (4) effects. In addition, many studies have also shown that rhein can improve the treatment of various diseases such as diseases of the nervous system (5), blood (6), digestive system (7, 8), metabolism (9), and kidney (10). In the study of liver injury, rhein has been found to help alleviate liver fibrosis caused by CCL4 (11), and for chronic liver injury caused by methotrexate (12) (MTX), rhein has been shown to inhibit the elevation and remission of alanine aminotransferase (ALT) and aspartate aminotransferase (AST) through the Nrf2-HO-1 pathway.

Acute liver injury is usually caused by viruses, drugs (13, 14), or autoimmune disorders and frequently leads to hepatocyte death. This type of injury is characterized by no underlying liver disease, rapid onset, and high mortality. At present, the main treatment for acute liver injury is liver transplantation. Although the survival rate of acute liver injury has been improved in recent years, its mortality rate remains high. Studies have found that acute liver injury caused by drugs (such as acetaminophen) (15) has a significantly higher survival rate without transplantation, and a large number of researchers have begun to conduct extensive investigations into nontransplantation treatment of acute liver injury.

In recent years, research on the liver-gut axis has gradually deepened, and the development of gut microbiome technology has led to the result that gut microbes are closely related to the occurrence and development of acute liver injury. Although probiotic strains primarily colonize the intestine, they can also interact with the liver at distant sites *via* the gut-liver axis and its associated metabolism (16). Studies have found that increasing intestinal probiotics such as Bacillus cereus (17), Bifidobacterium longum R0175 (18), and Lactobacillus reuteri DSM 17938 (19), can effectively reduce D-GaIN/LPS-induced increases in plasma AST and ALT, improve the abnormality of liver tissue, and regulate intestinal dysbiosis. Furthermore, studies have also found that the gut microbiome may regulate the level of acute liver injury through certain signaling pathways, such as with Lactobacilli (20), which can activate the transcription factor Nrf2 which has a protective effect on oxidative liver injury. Furthermore, Lactobacillus (21) is able to immunoregulate the recruitment of canonical dendritic cells (cDCs) to the liver to produce IL-10 and TGF-b *via* TLR9 activation, preventing further liver inflammation. However, studies have also shown that the gut microbiome can modulate the MYC pathway and exacerbate liver damage (22). Moreover, the metabolites of gut microbes such as 1-phenyl-1,2-propanedione (23) have been found to be present in increased proportions in the metabolites of gut microbiota in liver-injured mice, but Galactose, Myo-inositol and Oleic Acid (24, 25) metabolites have been found to be significantly lower in the gut microbiota of liver-injured mice.

In addition to the above, rhein has been reported to be able to modulate gut microbiota diversity and community composition, reduce obesity, and improve glucose tolerance in high-fat diet-fed rats (2). Not only that, but rhein can also regulate gut microbiota metabolism and relieve ulcerative colitis (26). We,therefore,hypothesize that gut microbiota is the “targets” for rhein in the treatment of acute liver injury.

## Materials and methods

### Animals and experimental design

Fifteen specific pathogen-free conventional male C57BL/6 mice weighing 18-20 g, were used for this study. After 1 week of adaptive feeding, we randomly divided the mice into three groups: the normal control group (NC group), the D-GaLN/LPS model group (Sigma-Aldrich; St. Louis, USA; cat: G0500/cat: L2630), and the rhein (Abmole Bioscience Inc: Catalog No: M3998) gavage treatment group, with five mice in each group. After 1 week of adaptive feeding, for the 8th to 15th days of the experiment, we gave rhein to the treatment group (100 mg/kg) by intragastric administration, and the other two groups were intragastrically administered the same dose of normal saline. On the 15th day of the experiment, the mice in the model group and rhein group were intraperitoneally injected with D-GalN/LPS (800mg/kg, 10ug/kg), and the normal control group was intraperitoneally injected with the same dose of physiological salt.

### Sample collection

After injection of D-GalN/LPS, all animals were anesthetized with ether 5 hours later, blood was collected from the eyeball for AST and ALT analysis, part of the liver was fixed with 4% paraformaldehyde for histomorphological analysis, and part of the liver was snap-frozen in liquid nitrogen for transcriptome analysis. Cecal contents were snap-frozen in liquid nitrogen and immediately stored at -80°C for further 16s rRNA and metabolomic analysis. During the experiment, all tissue samples were kept frozen for as long as possible.

### Assessment of liver injury

Plasma alanine aminotransferase (ALT) and aspartate aminotransferase (AST) were measured using an automatic biochemical analyzer (Chemray 800, rayto, China). We also excised 2 × 2 ×1 cm of liver tissue from the left lobe, fixed it with 4% formaldehyde, embedded it in paraffin, sectioned it, and stained it with HE. Pathological liver tissue damage was assessed using the HAI score (27).

### DNA extraction and 16S rRNA gene sequencing

The total genomic DNA of gut microbiota was extracted from feces using the E.Z.N.A.^®^ Soil DNA Kit (Omega Bio-Tek, USA). Then, DNA concentration and integrity were measured by NanoDrop2000 and agarose gel electrophoresis before sequencing preparation. The 27F_1492R region of the 16S rRNA gene of the microbiota was amplified using barcoded primers (forward primer: 27F: AGRGTTYGATYMTGGCTCAG; reverse primer: 1492R: RGYTACCTTGTTACGACTT) and sequenced using the Illumina MiSeq platform. A thermal cycler was performed on a PCR system (GeneAmp 9700, ABI, USA), and the mixture of PCR products was subsequently purified using an AxyPrep DNA Gel Extraction Kit. After purification, amplicons were combined in equal amounts and subjected to sequencing library preparation according to the manufacturer’s manual. Eligible libraries were sequenced on Pacbio Sequel II (Illumina, USA).

### Metabolite analysis

To analyze the metabolites of the gut microbiota, we first added 400 *μ*L of extraction solution (methanol:water=4:1(v:v), containing 0.02 mg/mL internal standard (L-2-chlorophenyl alanine) to 50mg fecal samples, ground them up, and extracted them by low-temperature ultrasonic extraction for 30 min (5°C, 40 KHz), let them stand at -20°C for 30 min, centrifuged them for 15 min (13000 g, 4°C), and pipetted the supernatant into a sample vial with an inner cannula for analysis on the computer.

Next, we performed LC-MS analysis and chromatographic separation on an ultra-high performance liquid chromatography-tandem Fourier transform mass spectrometry (UHPLC-Q Exactive HF-X) system. The chromatographic conditions were as follows. The column was ACQUITY UPLC HSS T3 (100 mm × 2.1 mm i.d., 1.8 *μ*m; Waters, Milford, USA), the column temperature was 40°C, and the flow rate was 0.4 mL/min. The mass spectrometry conditions were that the sample was ionized by electrospray and that the mass spectral signals were collected in positive (3500V) and negative ion (3500V) scanning modes, respectively, with a scanning range of 70-1050 (m/z), a sheath gas flow rate of 50 (arb), and an auxiliary gas flow rate of 13 (arb), a capillary temperature of 325°C, a heating temperature of 425°C, an S-Lens voltage of 50V, collision energy parameters of 20eV, 40eV, 60eV, and a resolution of 6000 (Full MS)/7500 (MS2). ProgenesisQI (Waters Corporation, Milford, USA) was used to perform baseline filtering, peak identification, integration, retention time correction, and peak alignment in order to screen for metabolic biomarkers that showed significant differences between different treatment groups. Finally, we matched the obtained precursor and fragment ions with metabolic databases: the human metabolome (database http://www.hmdb.ca/), and the Scripps database (https://metlin.scripps.edu/).

### RNA-seq analysis of the liver

Total RNA was extracted from tissue samples using the Zymo Quick-RNA™ Miniprep Kit (zymo), and we were able to isolate AT base pairings with polyA using magnetic beads with Oligo (dT). After this,we isolated mRNA isolated from total RNA, and this enriched mRNA was randomly broken into small fragments of 300bp. Next, the mRNA was used as a template to reverse synthesize one-strand cDNA using random primers, followed by two-strand synthesis. After adding adapters, the final library was obtained by purification and amplification, and qualified library 2X150bp duplex sequencing was performed on Illumina Novaseq. After filtering the raw data, high-quality sequencing data (clean data) was obtained for subsequent analysis. The original data after quality control and cleaning (reads) were then compared to the reference genome to obtain the number of read counts of genes, and then we carried out the expression difference analysis of genes between samples to identify genes that were differentially expressed between samples.

### Statistical analysis

Statistical analyses were performed using GraphPad 8.0. Data are presented as the Mean ± SEM. The differences between two groups were analyzed by Student’s t-test. Multiple group comparisons were analyzed using one-way analysis of variance (ANOVA) with Bonferroni correction. All results were considered statistically significant at P < 0.05. The differential metabolites were filtered by variable influence on projection (VIP) selection according to the PLS-DA and the filtering conditions VIP > 1 and P < 0.05. Spearman’s correlation values were computed with the R version 3.3.1. Metabolites were tentatively assigned by molecular formula matching and related information obtained from online databases such as the Human Metabolome Database (HMDB, http://www.hmdb.ca/spectra/ms/search) (28). Pathway analysis was performed on the KEGG website (29) (http://www.genome.jp/kegg/).

## Results

### The effects of rhein on acute liver injury caused by D-galactosamine/LPS

**Figure 1:**
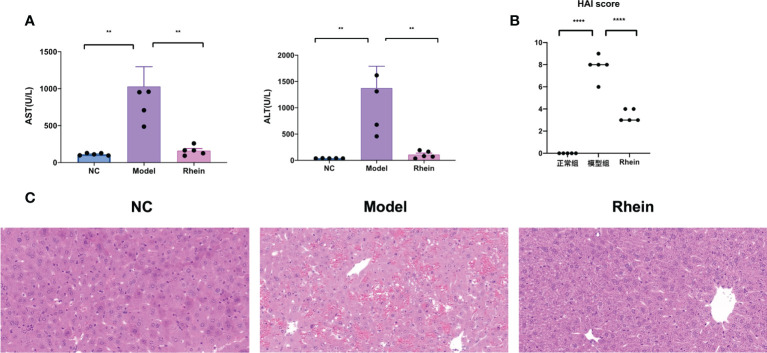
Rhein alleviates d-galactosamine/LPS-induced liver injury. **(A)** Plasma ALT and AST levels were determined 12 hours after intraperitoneal injection of D-GalN/LPS. **(B)** Liver HAI score. **(C)** Representative liver sections from different treatment groups (n = 6 each). (**P < 0.01, and ****P < 0.0001 compared to the ALF group, one-way ANOVA).

Compared to the normal control group, the plasma ALT and AST levels of the mice in the D-GalN/LPS group were significantly higher. Plasma ALT [NC vs Model, P < 0.01, Model vs Rhein, P < 0.01] and AST [NC vs Model, P < 0.01, Model vs Rhein, P < 0.001] levels were significantly relieved in the rhein group, however (**Figure 1A**). The histopathological results showed that there was no abnormal difference in the histological changes of the normal mouse liver; the hepatic lobules were clear, and no hepatocyte degeneration or necrosis was found (**Figures 1B, C**). In contrast, D-GalN/LPS treatment resulted in severe liver damage in the mice, but the rhein group showed significantly less liver damage and lower histological changes.

### The effects of rhein on the structure of intestinal flora

**Figure 2:**
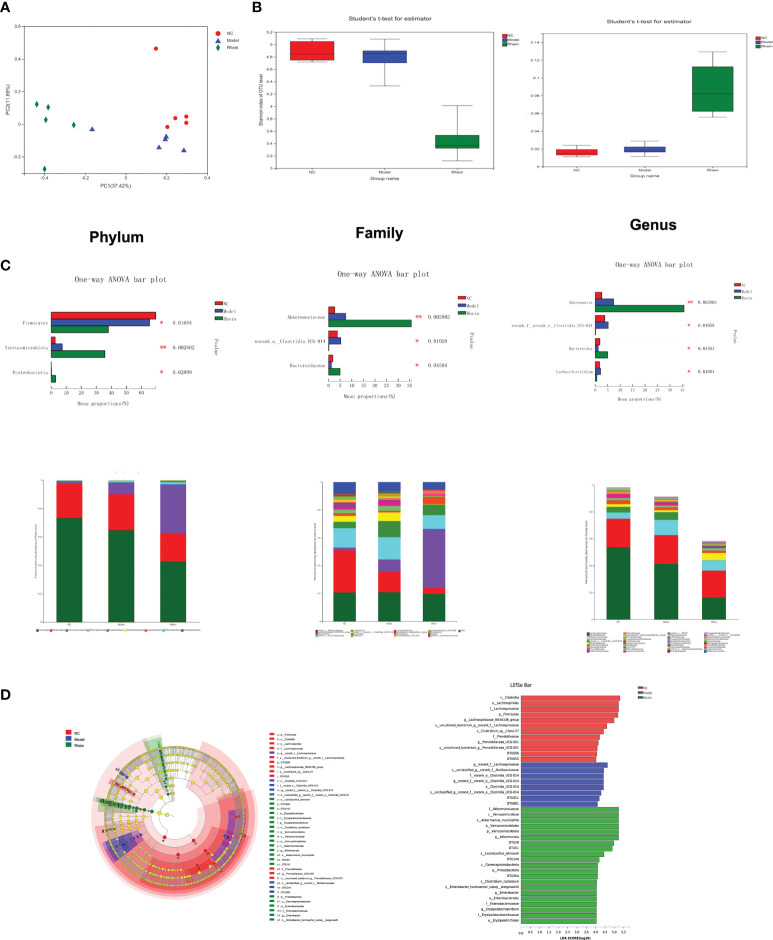
The effects of rhein on gut microbial composition and structure in mice (n =5). **(A)** Alpha diversity. **(B)** Beta diversity was determined by the Bray–Curtis distance-based principal. **(C)** predominant fecal microbial communities, and different bacteria at the phylum, family, and genus levels. (The symbol * indicates statistically significant differences between two groups, *p < 0.05 and **p < 0.01, one-way ANOVA). **(D)** The linear discriminant analysis effect size (LEfSe) analysis identified gut bacterial biomarkers in mice in three group.

Fecal samples were analyzed by 16s rRNA high-throughput sequencing technology to elucidate the regulatory role of rhein on gut microbiota, and we used the β-diversity analysis method to evaluate the diversity differences between different groups. The structure of gut microbiota was different in different treatment groups. Specifically, in our Bray–Curtis distance-based principal coordinate analysis (PCoA), the NC group showed a separation from the model group and the rhein group, and the gut microbiota structure of the model group showed distinct deviation from the rhein group (explaining 37.42% of the variation), indicating that the core microbiota changed significantly after treatment (**Figure 2A**). Using a diversity index to evaluate the diversity (Shannon index and Simpson index) of the microbial community (**Figure 2B**), we found that compared with the normal control group, the diversity of the microbiota of the mice in the model group decreased, and the intestinal microbes of the rhein intervention group decreased significantly.

The microbial community composition of mouse cecal contents showed that the microorganisms primarily consisted of Firmicutes (58.36%), Proteobacteria (24.92%), and Verrucomicrobiota (15.53%) at the phylum level (**Figure 2C**). Compared to the model group, the rhein intervention group had higher levels of Verrucomicrobiota (P<0.01) and Proteobacteria (P<0.05). Additionally, the most abundant families included Lachnospiraceae (42.52%), Muribaculaceae (22.20%), Akkermansiaceae (17.02%), Lactobacillaceae (7.62%), Ruminococcaceae (2.41%), Bacteroidaceae (1.74%), and Oscillospiraceae (1.35%). The amount of Norank_o:Clostridia_UCG-014 in the model group was higher than that in the normal control group and rhein group (P<0.05), but Akkermansiaceae (P<0.01) and Bacteroidaceae (P<0.05) in the rhein treatment group were much higher than those in the model group. The most abundant genera were norank_f:Muribaculaceae (23.96%), Akkermansia (18.88%), Lachnospiraceae_NK4A136_group (17.79%), norank_f:Lachnospiraceae (14.73%), Lactobacillus (8.45%), Bacteroides (1.94%), Lachnoclostridium (1.54%), Roseburia (1.46%), Lachnospiraceae_UCG-006 (1.09%), and Mucispirillum (1.07%). Compared to the normal control group and the rhein group, the relative abundances of Lachnoclostridium and norank_o:Clostridia_UCG-014 in the model group were higher (P<0.05). Furthermore, the abundance of Roseburia in the model group was also higher than that in the other two groups, but the difference was not statistically significant. Finally, compared to the model group, the abundance of Bacteroides in the rhein group was higher (P<0.05).

Our LEfSe analysis further showed the abundance of differential taxa, and a histogram with logarithmic LDA > 4.0 and a cladogram is shown in **Figure 2D**. This analysis showed that acute liver injury was accompanied by higher amounts of o:Clostridia_UCG-014, f:norank_o:Clostridia_UCG014, and g:norank_f:norank_o:Clostridia_UCG-014 and that the rhein intervention group was more abundant in p:Verrucomicrobiota, c:Verrucomicrobiae, o:Verrucomicrobiales, f:Akkermansiaceae, and g:Akkermansia, :Johns:Akkeracactii.

### The effects of rhein on intestinal metabolism

**Figure 3:**
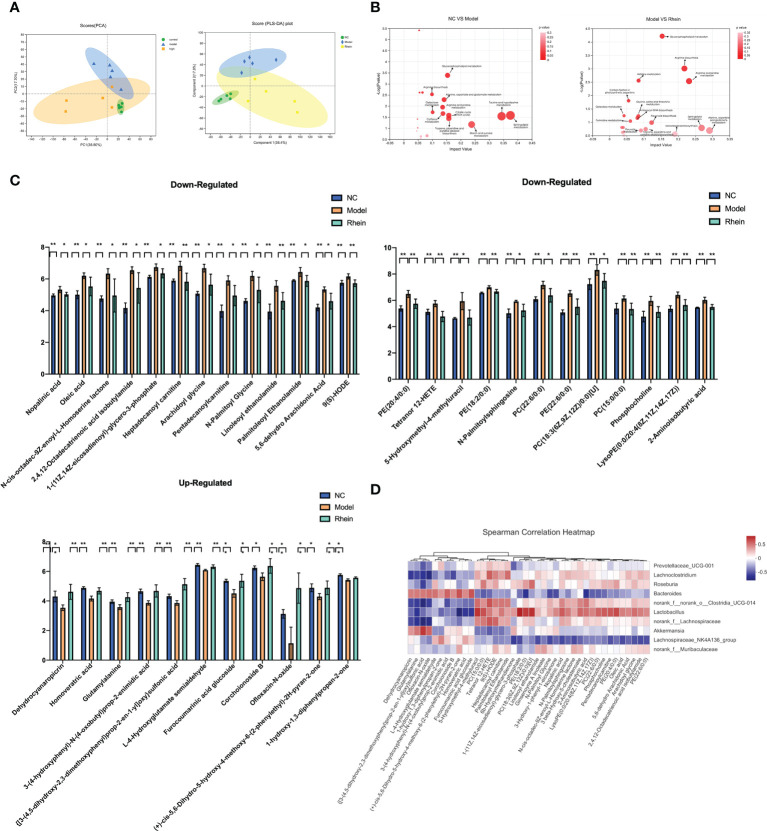
Rhein protects against D-galactosamine/LPS-induced altered feceal metabolites. **(A)** Principal component analysis (PCA) (PC1 = 38.8%): the partial least squared discriminant analysis (PLS-DA) score plot based on LC-MS profiling data of feceal samples (compound1 = 47.2%). Each dot with three kinds of color represents the different samples. **(B)** Meaningful metabolic pathways in the comparison of NC and Model group, and Model and Rhein groups. **(C)** Based on VIP > 1, P < 0.05, FDR < 0.05 as a filter for differential metabolites between normal control and ALF groups, and rhein treatment significantly improved metabolic disorders, *p < 0.05 and **p < 0.01. **(D)** Heatmap of the correlation between the altered microbial community and significantly changed metabolites. The color indicates the Spearman’s correlation coefficient.

The complex interactions between the host and gut microbiota are closely related to the host-microbe metabolic axis. Hence, we next performed untargeted metabolomics of stool samples using liquid chromatography-mass spectrometry (LC-MS). Overall there were 997 and 869 metabolites identified in feces, under the negative and positive modes, respectively. The score plots of principal component analysis PCA (38.80%) and partial least squares discriminant analysis OPLS-DA (38.40%) showed that the metabolome profiles of the NC group, Model group, and rhein group were clustered separately (**Figure 3A**). There were significant differences in metabolic profiles between the three groups.

In analyzing the different metabolites among the three groups, we detected a total of 818 differential metabolites. We conducted KEGG pathway enrichment analysis for all differential metabolites (**Figure 3B**) and found that compared to the normal group, the most significant metabolic pathways in the model group were taurine and hypotaurine metabolism, sphingolipid metabolism, starch and sucrose metabolism, the citrate cycle (TCA cycle), tropane, piperidine and pyridine alkaloid biosynthesis, glycerophospholipid metabolism, alanine, aspartate and glutamate metabolism, caffeine metabolism, arginine and proline metabolism, galactose metabolism, and arginine biosynthesis.

Pathways of differential metabolites in the rhein treatment group and acute liver injury group had higher amounts of alanine, aspartate and glutamate metabolism, sphingolipid metabolism, arginine and proline metabolism, arginine biosynthesis, monoterpenoid biosynthesis, glycerophospholipid metabolism, flavonoid biosynthesis, tropane, piperidine and pyridine alkaloid biosynthesis, pyruvate metabolism, histidine metabolism, glycine, serine and threonine metabolism, aminoacyl-tRNA biosynthesis, pyrimidine metabolism, carbon fixation in photosynthetic organisms, and galactose metabolism. This suggests that the rhein group may have partially reversed some of the side effects of D-GalN/LPS through these metabolisms.

Next, to discover the possible biomarkers of rhein treatment, we used Student’s *t*-test to compare metabolite variation in acute liver injury between the three groups. We found that 224 metabolites were significantly changed between the normal control group and the model group (VIP > 1, P < 0.05, FDR < 0.05). Among them, 126 metabolites gradually returned to normal after rhein treatment, and 36 metabolites had statistical significance (P<0.05). Among these, the rhein group up-regulated 11 metabolites reduced by D-GalN/LPS and down-regulated 25 other metabolites (**Figure 3C**). Our spearman correlation analysis of microbiota and metabolites found correlations between the top 40 significantly altered differential fecal metabolites and the top 10 most abundant gut microbes (**Figure 3D**). We found that Dehydrocyanaropicrin, Homoveratric acid, Glutamylalanine, 3-(4-hydroxyphenyl)-N-(4oxobutyl)prop-2-enimidic acid, {[3-(4,5-dihydroxy-2,3-dimethoxyphenyl)prop- 2-en-1yl]oxy}sulfonic acid, L-4-Hydroxyglutamate semialdehyde, furocoumarinic acid glucoside, corchoionoside B, Ofloxacin-N-oxide, (+)-cis-5,6-Dihydro-5-hydroxy-4 -methoxy-6-(2-phenylethyl)- 2H-pyran-2-one, and 1-hydroxy-1,3-diphenylpropan-2-one were all positively correlated with Bacteroidesfen but negatively correlated with Lachnoclostridium, norank_o:Clostridia_UCG-014. dehydrocyanaropicrin, glutamylalanine, {[3-(4,5-dihydroxy-2,3-dimethoxyphenyl)prop-2-en-1yl]oxy}sulfonic acid, and ofloxacin-N-oxide were also positively correlated with Akkermansia but negatively correlated with Roseburia Related. Finally, 5-hydroxymethyl-4-methyluracil, PC(15:0/0:0), Tetranor, 12-HETE, 9(S)-HODE, Heptadecanoyl, Carnitine, Paln, litoleoyl Ethanolamide showed positive correlation with Lachnoclostridium, norank_o:Clostridia_UCG-014, and Roseburia Positive correlation but negative correlation with Bacteroidesfen and Akkermansia.

### The effects of rhein on mouse liver genes

**Figure 4:**
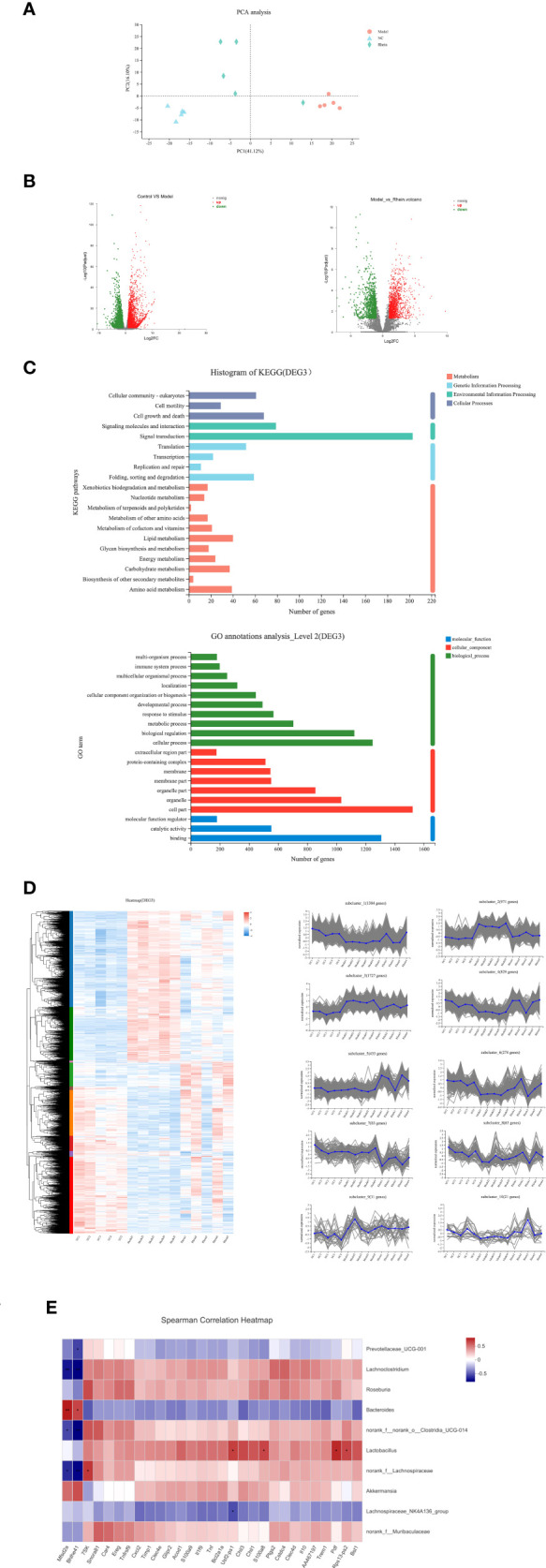
Comparisons of gene expression profiles in the mouse livers by RNA-seq among different treatment groups. **(A)** Principal component analysis showed that the Model group had distinct gene expression signatures compared with the NC group and rhein group PC1 (explaining 77.68% of variation). **(B)** Compared to the NC group, the number of differentially expressed genes (DEGs) in the Model groups was 5,220 (DEG1 2,663/2,557, upregulated and downregulated DEGs, DEGseq, respectively). Compared to the Model group, the number of differentially expressed genes (DEGs) in the rhein group was 2,503 (DEG2, 1,499/1,004, up-regulated and down-regulated DEGs, DEGseq, respectively). **(C)** The number of genes for Rhein vs. ALF and NC vs. ALF with the same trend was 1,907 (DEG3): KEGG annotation of DEG3 and GO annotation analysis of DEG3. **(D)** Heatmap of DEG3. DEG3 genes were further clustered into 10 Genes clusters according to their expression profiles. **(E)** Heatmap of the correlation between the altered microbial community and significantly changed gene expressions. *P < 0.05, **P < 0.01.

We used transcriptome analysis to determine whether the gene expression profiles of mouse livers were similar between different treatment groups. Principal component analysis showed that the genes of the mice had significant segregation (41.12%), and compared to the distance between the rhein group and the normal group, the mice in the model group and the normal group had more obvious segregation (**Figure 4A**). We then identified differentially expressed genes (FC >2 or less than 0.5, Padj < 0.05) by pairwise comparison of groups. Compared to the normal group, the number of differentially expressed genes (DEGs) in the ALF group was 5,220 (DEG1 2663/2557; up-regulated and down-regulated DEGs and DEGseq), and compared to the ALF group, the number of differentially expressed genes (DEGs) in the rhein group was 2,503 (DEG2, 1499/1004; up-regulated and down-regulated DEGs and DEGseq) (**Figure 4B**). Among these, rhein vs. ALF and NC vs. ALF intersected for 1,907 genes (DEG3), indicating that rhein may alleviate acute liver injury through these genes. Next, DGE3 underwent KEGG annotation and Gene Ontology (GO) term annotation analysis, and our KEGG annotation of significantly differentially expressed genes indicated that most genes were annotated to amino acid metabolism, carbohydrate metabolism, energy metabolism, lipid metabolism; folding, classification and degradation, transcription, translation, signal transduction, signaling molecules and interactions, cell growth and death, cell movement, cell communities, and eukaryotes. Our GO annotations primarily involved molecular functions, cellular organization, and biological processes (**Figure 4C**).

In order to study the expression of rhein vs. ALF and NC vs. ALF for the intersection gene DEG3 in each group, we generated related expression heatmaps and further aggregated these into 10 cluster trend maps (**Figure 4D**). Compared to the NC group and rhein group, subcluster1 and subcluster6 in the ALF group showed an activated expression pattern, while subcluster2, subcluster4, subcluster7, subcluster8, and subcluster10 in the ALF group were inhibited compared to the NC group and rhein group. Next, we further screened the activated and inhibited gene groups in the model group (5<Log2FC<5, Padjust<0.05). In the model group, 9 genes were down-regulated (**Table 1**), and 102 genes were up-regulated (**Table 2**), which were recovered after rhein treatment (P<0.05). For our correlation analysis between transcriptome genes and microbiota, we selected the top 30 genes with the largest differences in the above-mentioned gene table with significant differences for correlation analysis with the top 10 microorganisms in the gut microbiota (**Figure 4E**). These results showed that Mfsd2a and Bhlhe41 genes were positively correlated with Bacteroides (P<0.001; P<0.05) and that they were also positively correlated with Akkermansia, but this correlation was not statistically significant. The other 28 genes, including Cxcl2, S100a9, Tnf, Ereg and IL-10 were activated genes in the model group and were associated with Lachnoclostridium, Roseburia, norank_f:norank_o:Clostridia_UCG-014, Lactobacillus, norank_f:Lachnospiraceae, and norank_f:Muribaculaceae, which were all positively regulated.

**Table 1:** Significantly down- regulated genes (-5<Log2FC<5, Padjust<0.05) upon exposure of D-DalN/LPS compared to the control group.

Gene name	Log2FC (Model/NC)	Padjust (NC/Model)	Padjust (Model/Rhein)
Mfsd2a	-8.107638231	0.00	0.00
Bhlhe41	-6.766796975	0.00	0.01
Per3	-5.665128898	0.00	0.01
Fam13a	-5.624820496	0.00	0.02
Grem2	-5.220023357	0.03	0.00
Bbs10	-5.171843892	0.00	0.01
Thsd1	-5.104965618	0.00	0.05
Zfp108	-5.020511277	0.00	0.05
Ttc30b	-5.00163948	0.00	0.00

The genes significantly recovered after rhein treatment (Padjust<0.05).

**Table 2:** Significantly up-regulated genes (-5<Log2FC<5, Padjust<0.05) upon exposure of D-DalN/LPS compared to the control group.

Gene name	Log2FC (Model/NC)	Padjust (NC/Model)	Padjust (Model/Rhein)
Car4	6.919175284	0.00	0.00
Timp1	6.944975523	0.00	0.04
Tgfb1	5.151649063	0.00	0.00
Ier3	5.506764099	0.00	0.00
Hmox1	5.212161173	0.00	0.00
Adamts4	6.706936815	0.00	0.03
Ccl8	5.324963207	0.00	0.00
Stc1	6.06811393	0.00	0.03
Slamf1	5.655920857	0.00	0.00
Il10	6.751278575	0.00	0.00
Cd40	6.349012026	0.00	0.03
Adora2b	5.320542683	0.00	0.01
Plek	6.476748845	0.00	0.00
Adora2a	5.050565758	0.00	0.02
Gfpt2	5.366385622	0.00	0.01
Pik3r5	5.153700574	0.00	0.00
Arg2	6.261374572	0.00	0.00
Acod1	10.4080818	0.00	0.05
Samsn1	6.538537579	0.00	0.00
Cdkn1a	6.453109266	0.00	0.04
Gzma	5.062957775	0.00	0.00
Apom	5.116050176	0.00	0.01
Tnf	7.491073348	0.00	0.00
Hbegf	6.132634273	0.00	0.00
Ms4a4c	5.40723121	0.00	0.01
Ms4a6d	5.985060818	0.00	0.00
Slc16a3	6.097855153	0.00	0.00
Il6	6.740192438	0.00	0.01
Ccr1	5.242404986	0.00	0.00
Sema4c	6.110668584	0.00	0.02
Gpr35	6.249355827	0.00	0.00
Ifi211	6.64611414	0.00	0.01
Sell	5.184631989	0.00	0.00
Il1b	5.774572407	0.00	0.00
Procr	6.322937193	0.00	0.00
Glipr2	7.662069079	0.00	0.03
Tnfrsf9	7.222147271	0.00	0.00
Bst1	7.000658331	0.00	0.00
Ppbp	6.533548869	0.00	0.00
Ereg	7.283104436	0.00	0.01
Clec4e	7.841833483	0.00	0.00
Clec4d	7.743163983	0.00	0.00
Olr1	6.171770662	0.00	0.04
Pglyrp1	6.041945188	0.00	0.00
Siglece	5.451987043	0.00	0.00
Adm	5.451046818	0.00	0.00
Il2rg	5.922866163	0.00	0.01
Angpt2	5.046610524	0.00	0.00
Ngp	6.211127623	0.00	0.00
Ptgs2	7.49760651	0.00	0.00
AA467197	7.869514992	0.00	0.00
Batf	6.738098848	0.00	0.00
Jdp2	5.270912884	0.00	0.03
Dbn1	6.522036342	0.00	0.00
Ccl5	5.736058999	0.00	0.01
Serpine1	6.105035149	0.00	0.00
Egr2	6.365133619	0.00	0.00
Slamf7	5.910760118	0.00	0.00
Batf2	5.665438455	0.00	0.02
Spsb1	5.327076278	0.00	0.02
Chil3	8.006701052	0.00	0.00
Serpina3g	5.669202334	0.00	0.03
Trem3	5.377878513	0.00	0.03
Trem1	6.934995142	0.00	0.00
Ccno	5.265875138	0.00	0.00
Il1f9	8.283161225	0.00	0.01
Fpr1	5.057008415	0.00	0.00
Plaur	5.781148943	0.00	0.01
Marcksl1	5.065599451	0.00	0.02
Selplg	5.380248123	0.00	0.00
Tmem252	5.608693398	0.00	0.03
Rasd1	5.127485541	0.00	0.00
Lrrc25	5.036998147	0.00	0.01
Fpr2	5.34870867	0.00	0.00
Hbb-bs	6.318989389	0.00	0.00
S100a8	7.009760337	0.00	0.02
S100a9	7.779229798	0.00	0.00
Capg	5.48152647	0.00	0.00
Cxcl2	9.817770623	0.00	0.00
Osm	5.163056101	0.00	0.04
Nfe2	5.860432292	0.00	0.02
Fcgr4	5.007453977	0.00	0.00
Chil1	7.752737989	0.00	0.01
Snora73a	5.780667664	0.00	0.00
Hba-a2	5.89064318	0.00	0.00
Hba-a1	5.840920595	0.00	0.00
Rps13-ps2	7.199941268	0.00	0.00
Sp110	5.580940347	0.00	0.04
Hbb-bt	5.972566189	0.00	0.00
Pdf	7.367754009	0.00	0.00
Ms4a6c	5.06313634	0.00	0.00
Cstdc4	7.270625276	0.00	0.02
Dnah2os	5.722701063	0.00	0.00
Snora81	7.677871672	0.00	0.03
Apold1	5.765146149	0.00	0.00
Ms4a4a	6.453485429	0.00	0.00
Bcl2a1a	7.012518761	0.00	0.04
Usf2-ps1	7.124584422	0.00	0.00
7SK	7.365687369	0.00	0.01

The genes significantly recovered after rhein treatment (Padjust<0.05).

## Discussion

Our main finding is that the group pretreated with rhein had substantially better outcomes from liver damage after D-GalN/LPS injection. Additionally, we observed several other beneficial effects in the rhein group, including attenuation of microbial dysbiosis, improvement of metabolic profile, and modulation of certain gene levels. We also summarized the results of our 16S rRNA gene sequencing metabolomic and transcriptomic analysis and discussed the relationship between rhein and acute liver injury in light of the association of gut bacteria with metabolic biomarkers and liver tissue genes.

Serum ALT and AST levels have been shown to be the major biomarkers for liver injury (30), and this study showed that the rhein group had significantly lower D-GalN/LPS-induced AST, ALT elevation, and liver tissue damage than the other two groups. The changes in these two functional indices, along with the improvement in HAI score, indicated that the hepatocyte injury in the rhein group was alleviated compared to the model group. We also applied fecal microbiome sequencing to identify changes in the microbiota and found that Lachnoclostridium, norank_o:Clostridia_UCG014 and Roseburia were higher in the gut of mice during acute liver injury, whereas Bacteroides and Akkermansiacea were lower in the acute liver injury group. Among these, Lachnoclostridium has been found to be significantly more abundant in the gut of high-fat diet rats (31, 32) but was significantly inhibited after our treatment. Roseburia has also been shown to increase in abundance in high-fat diet mice and has been found to be positively correlated with increased deoxycholic acid in the plasma, liver tissue, and feces of high-fat diet mice as well (33). Studies have shown that deoxycholic acid is a cytotoxic bile acid that can activate oxidative stress and promote hepatocyte apoptosis, causing various diseases of the liver (34, 35).

Additionally, norank_o:Clostridia_UCG-014 is associated with ulcerative colitis, and it was significantly higher in the mice in our model group. In models with a tendency to self-heal, this bacteria has shown a downward trend (36). However, our rhein group showed signs of regulated DGalN/LPS-induced gut flora disturbance and suppressed abundance of Lachnoclostridium, norank_o:Clostridia_UCG-014 and Roseburia, and higher amounts of Bacteroides and Akkermansiacea. We further validated these results by LEfSe analysis and found that two species, Akkermansia_muciniphila and Lactobacillus_johnsonii, dominated the rhein-treated group. Akkermansiacea and Bacteroidesfen have been found to be lower in the feces of patients with cirrhosis and nonalcoholic fatty liver, respectively, and both have been shown to be negatively correlated with elevated calprotectin concentration in the feces of patients with cirrhosis (37).

Akkermansia_muciniphila has recently been recognized as a next-generation probiotic strain for the treatment of obesity-related diseases (38), and studies have found that supplementation with Akkermansia_muciniphila can reduce the levels of blood markers related to liver dysfunction and inflammation (39) and improve oxidative stress-induced intestinal Apoptosis (40), reduce neutrophil infiltration (41), maintain intestinal barrier function, and promote of short-chain fatty acid (SCFA) secretion (42) as well, thereby remodeling the composition of gut microbiota. It also has preventive effects on fatty liver (40), alcoholic liver disease (41), and hepatic fibrosis induced by acetaminophen or carbon tetrachloride (42, 43), and it has s extracellular vesicles (EVs), which are the cell membranes of Gram-negative and Gram-positive bacteria that can interact not only with host cells but also with other microbiota. Akkermansia_muciniphila has also been found to improve intestinal permeability, modulate inflammatory responses, and prevent liver injury in HFD/CCl4-administered mice (44, 45).

Lactobacillus_johnsonii is a type of lactobacillus that adheres to intestinal cells (46). Studies have shown that it can inhibit the cell adhesion of toxic bacteria in the intestine and help maintain intestinal microecology. In addition, Lactobacillus_johnsonii has been shown to improve bacterial translocation in cirrhotic rats with ascites, and bacterial translocation is closely related to mucosal oxidative damage and impaired intestinal permeability (47, 48). Therefore, we speculate that rhein can regulate the intestinal microbiota, induce a more favorable composition of intestinal microbiota, improve the intestinal barrier, and improve inflammatory and oxidative stress responses to prevent and treat acute liver injury.

In the primary differential metabolic-pathway-amino-acid metabolism, a number of differential metabolites were screened in this study, among which Tryptophanol, N-acetylputrescine, L-Glutamine, N-Aarbamoylsarcosine, 2-Hydroxycinnamic acid, Ornithine, Citrulline, Maleic acid, L-Proline and L-Aspartic acid were down-regulated in the rhein treatment group. In addition, the rhein group showed up-regulated 3-Indoleacetic acid, Formiminoglutamic acid, L-Arogenate, Vanillylmandelic Acid, Stizolobate, Allysine, Oxoglutaric acid, Indole Acetaldehyde, Gentisic acid, 5hydroxyindoleacetaldehyde, 2-Isopropylmalic acid, Pipecolic acid, Imidazole acetic acid, acid riboside, L- 4-hydroxyglutamate semialdehyde, Citric acid, and Phenyl lactic acid. We consider these to be biomarkers that can be used to assess the effects of rhein treatment on fecal metabolites in mice.

There were multiple metabolites that were significantly different between the normal control group and the model group but that returned to normal levels after rhein treatment. Among the metabolites up-regulated in the rhein group, L-4-hydroxyglutamate semialdehyde in the above-mentioned biomarkers is an organic compound of L-alpha-amino acids, and studies have found that amino acid metabolism disorders play an important role in the pathological process of drug-induced liver injury. *In vitro* experiments (49, 50) have found that L-alpha-amino acids can participate in the consumption of TAMa• free radicals, which are stable carbon-centered free radicals that are mostly consumed by oxidative metabolism in liver microsomes. Experimental results have shown that the consumption of TAMa• free radicals by L-alpha-amino acids is similar to that of glutathione.

Abnormal amino acid metabolism, such as L-tyrosine and taurine, has been found to be associated with hydrazine-induced liver injury *in vivo* (51). In our experiment, two antibacterial components, ofloxacin-N-oxide and 1-hydroxy-1,3-diphenylpropan-2-one, in the up-regulated metabolism of the rhein group were also significantly higher in the fecal metabolites of these mice compared to the model group. Ofloxacin-N-oxide, a metabolite of Ofloxacin, has antibacterial effects on 150 pathogens such as Enterobacteriaceae and Haemophilus influenzae, and can produce antibacterial activity against some pathogens in patients with severe diseases (52). Similarly, 1-hydroxy-1,3diphenylpropan-2-one has shown antibacterial activity against 13 strains of methicillin-resistant Staphylococcus aureus (MRSA) (53). In this study, the rhein group down-regulated multiple metabolites related to liver diseases, such as tetranor 12-HETE, which is significantly associated with nonalcoholic fatty liver fibrosis and can be used as a noninvasive biomarker of liver fibrosis (54) and 9(S) –HODE, which is an endogenous fatty acid (PPAR) gamma agonist and is closely related to hepatic steatosis. After dietary intervention in obese adolescents, 9-HODE, ALT, triglyceride, and cholesterol levels have been found to be significantly reduced (55–57). So the metabolites may jointly improve acute liver injury through some direct and indirect pathways, including inhibiting harmful flora in the gut, regulating the disorder of metabolites such as amino acids, and participating in the oxidative metabolism of free radicals.

We also analyzed liver gene expression in different groups of mice. The model group mice had different gene expression profiles compared to the normal group mice, and the rhein treatment group had significantly altered liver gene expression by up-regulating and down-regulating specific gene groups, with more similar gene expression to the normal control group. The GO and KEGG pathways that were significantly enriched in the rhein pretreatment group were similar to those of the metabolomics results and were mostly those responsible for regulating amino acid, lipid, and carbohydrate metabolism. This was also consistent with the metabolomic results from the mouse feces.

Research has shown that rhein is a potential treatment for inflammatory diseases (58, 59), and cancer, and we found evidence for its anti-inflammatory, anti-oxidative (60), and anti-cancer effects in this study as well. The model group was enriched with genes that were positively related to Lachnoclostridium and norank_o:Clostridia_UCG-014, and the genes’ functions were mostly proinflammatory, pro-apoptotic, and cancer-promoting, aggravating the degree of liver damage. For example, CXCL2 can recruit neutrophils to help with immunity, induce immunosuppression, and promote HCC progression (61), and S100A9 levels have been shown to play a role in liver necroinflammation and necroptosis (62, 63). Furthermore, the Tnf gene can induce multiple mechanisms to initiate hepatocyte apoptosis, leading to subsequent liver injury (64), and Ereg and IL10 are up-regulated in acute liver injury and hepatocellular carcinoma, respectively (65, 66). however, the mice in the rhein group not only down-regulated the above genes that have a positive effect on liver injury, but also the genes that are positively related to enriched gut flora, that have anti-cancer properties, and that promote liver regeneration. For example, MFSD2A is known to help maintain the blood-brain barrier (67). Recent studies have found that it may also act as a new tumor-suppressing gene in regulating the cell cycle, and it plays an important role in matrix attachment as well (68).

Experimental results have also shown that the mRNA and protein levels in cancer tissues are significantly lower than those in adjacent normal tissues (69, 70). Other studies have shown that MFSD2A+ is expressed in many tissues (especially in the liver) and is not only significantly downregulated in hepatocellular carcinoma but also able to repopulate the liver during hepatocyte regeneration (71). In addition, researchers have found that Bhlhe41 is negatively correlated with the transcriptional repressor capicua (CIC) (72) and that CIC is involved in immune regulation. When CIC is inhibited, it can promote follicular helper T (Tfh) and liver-resident memory-like CD8+. The differentiation of T cells (73, 74), both of which are important cell groups in human immunity, maintain the immune balance of the body, and studies have found that there is a causal relationship between the occurrence of immunity and cancer (75). Rhein may up-regulate anti-cancer and liver regeneration-promoting genes, and down-regulate pro-inflammatory, pro-apoptotic, and pro-oncogenes through intestinal flora, and may also alleviate acute liver injury caused by D-GalN/LPS.

## Conclusion

Our results suggest that our rhein treatment alleviated D-GalN/LPS-induced acute liver injury in mice, improved intestinal flora disturbance, and modulated metabolic abnormalities and gene expression. From the perspective of gut microbes, we find that rhein may be able to help prevent and treat acute liver injury. Bacteroides and Akkermansiacea may have certain therapeutic effects on acute liver injury. Lachnoclostridium, norank_o:Clostridia_UCG-014 and Roseburia may have some exacerbating effects of acute liver injury. In addition, we described the relationship between microbiota and metabolites and microbiota and gene expression and found that gut microbiota is correlated with a variety of amino acid metabolites and gene expressions for immunity, apoptosis, and cancer.

In the future, we will conduct further experimental studies on the mechanism of rhein in alleviating acute liver injury. And we will continue to improve the investigation of the correlation between rhein and intestinal flora and carry out experimental verification such as flora transplantation, to clarify whether the intestinal flora and metabolites regulated by rhein have an certain protective effect on acute liver injury.

## Data availability statement

The data presented in the study are deposited in the NCBI repository, accession number PRJNA891652, PRJNA892154.

## Ethics statement

The animal study was reviewed and approved by Capital Medical University.

## Author contributions

SL, JH, and FW conceived and designed the experiments. JH, FW, SL, RY, and ZY were involved in the experimental study design, preparation, and review of this manuscript. All authors contributed to the article and approved the submitted version.

## Funding

This research was funded by the Key medical major of Beijing sailing plan, severe liver disease with integrated traditional Chinese and Western medicine (No. zylx201819).

## Acknowledgments

The authors thank AiMi Academic Services (www.aimieditor.com) for the English language editing and review services.

## Conflict of interest

The authors declare that the research was conducted in the absence of any commercial or financial relationships that could be construed as a potential conflict of interest.

## Publisher’s note

All claims expressed in this article are solely those of the authors and do not necessarily represent those of their affiliated organizations, or those of the publisher, the editors and the reviewers. Any product that may be evaluated in this article, or claim that may be made by its manufacturer, is not guaranteed or endorsed by the publisher.
